# Mitogenomes of Two *Phallus* Mushroom Species Reveal Gene Rearrangement, Intron Dynamics, and Basidiomycete Phylogeny

**DOI:** 10.3389/fmicb.2020.573064

**Published:** 2020-10-23

**Authors:** Cheng Chen, Jian Wang, Qiang Li, Rongtao Fu, Xin Jin, Wenli Huang, Daihua Lu

**Affiliations:** ^1^Institute of Plant Protection, Sichuan Academy of Agricultural Sciences, Chengdu, China; ^2^Key Laboratory of Integrated Pest Management on Crops in Southwest, Ministry of Agriculture, Chengdu, China; ^3^School of Food and Biological Engineering, Chengdu University, Chengdu, China; ^4^Biotechnology and Nuclear Technology Research Institute, Sichuan Academy of Agricultural Sciences, Chengdu, China

**Keywords:** *Phallus*, mitochondrial genome, intron, gene rearrangement, phylogenetic analysis

## Abstract

*Phallus indusiatus* and *Phallus echinovolvatus* are edible bamboo mushrooms with pharmacological properties. We sequenced, assembled, annotated, and compared the mitogenomes of these species. Both mitogenomes were composed of circular DNA molecules, with sizes of 89,139 and 50,098 bp, respectively. Introns were the most important factor in mitogenome size variation within the genus *Phallus*. *Phallus indusiatus*, *P. echinovolvatus*, and *Turbinellus floccosus* in the subclass Phallomycetidae have conservative gene arrangements. Large-scale gene rearrangements were observed in species representing 42 different genera of Basidiomycetes. A variety of intron position classes were found in the 44 Basidiomycete species analyzed. A novel group II intron from the *P. indusiatus* mitogenome was compared with other fungus species containing the same intron, and we demonstrated that the insertion sites of the intron had a base preference. Phylogenetic analyses based on combined gene datasets yielded well-supported Bayesian posterior probability (BPP = 1) topologies. This indicated that mitochondrial genes are reliable molecular markers for analyzing the phylogenetic relationships of the Basidiomycetes. This is the first study of the mitogenome of the genus *Phallus*, and it increases our understanding of the population genetics and evolution of bamboo mushrooms and related species.

## Introduction

Mitochondria are organelles in eukaryotes that may have originated from symbiotic bacteria. Mitochondria contain genetic information that differs from the information in nuclear genomes (Muñoz-Gómez et al., 2017). Mitochondria dysfunction creates problems in energy metabolism, aging, and disease (Shigenaga et al., 1994; Mcfarland et al., 2007). The mitochondrial genome (mitogenome) has been used in evolution, phylogeny, and population genetic studies because of its advantages of matrilineal inheritance, small size, conserved gene sequences, and a high mutation rate (Paquin et al., 1997; Joardar et al., 2012). Next-generation sequencing technology has enabled the analysis of many mitogenomes. However, the mitogenomes of fungi are less studied than those of animals (Chatre and Ricchetti, 2014). In particular, the number of Basidiomycete mitogenomes determined is far fewer than the research needs of this group (Li et al., 2019a). Fungal mitogenomes typically contain 14 conserved protein-coding genes (PCGs) (*atp6*, *atp8*, *atp9*, *cob*, *cox1*, *cox2*, *cox3*, *nad1*, *nad2*, *nad3*, *nad4*, *nad4L*, *nad5*, and *nad6*), one ribosomal protein S3 gene (*rps3*), two ribosomal RNA genes (*rnl* and *rns*), and a relatively constant set of tRNA genes (Kang et al., 2017). In addition, homing endonuclease genes, plasmid-derived genes, genes transferred from the nuclear genome, and some unknown functional genes have been found in the mitogenomes of different species of fungi. The mitochondrial genes, introns, and intergenic regions in fungi lead mitogenome sizes ranging from 18.84 kb (*Hanseniaspora uvarum*) to 272.24 kb (*Morchella importuna*) (Liu et al., 2020). Different gene arrangements, structures, and intron losses and gains occur in the mitogenomes of different fungal species, even in those of congeners (Brankovics et al., 2017; Deng et al., 2018b). Analysis of the composition and variation of the mitogenomes of different species can help reveal their phylogeny and evolutionary relationships.

*Phallus* species commonly known as stinkhorn fungi are widely distributed saprophytic mushrooms (Verma et al., 2019). *Phallus indusiatus* and *Phallus echinovolvatus* (Synonym, *Dictyophora indusiata* and *Dictyophora echinovolvata*, Chinese name Zhu Sun, commonly called bamboo mushrooms) are edible mushrooms with medicinal properties (Deng et al., 2018a). *Phallus indusiatus* has a cosmopolitan distribution in the tropics and subtropics, including southern Asia, Africa, Australia, and the Americas (Bandala et al., 1999). *Phallus indusiatus* fruiting bodies, and their polysaccharide components, have immunoregulatory (Hua et al., 2012; Liao et al., 2015), antioxidative (Nguyen et al., 2013), anti-inflammatory (Nguyen et al., 2013), antineoplastic (Deng et al., 2013), neuroprotective (Zhang et al., 2016), antihyperlipidemic, and hepatoprotective (Wang et al., 2019) activities. *Phallus echinovolvatus* is widely cultivated in China because of its strong resistance to high temperatures and drought and its pharmacological activity (Yu et al., 2017). The two species differ in the size and shape of the indusium (a delicate lacy “skirt” that hangs beneath the cap) (Shi et al., 2019). The presence and characteristics of the indusium are important taxonomic characteristics that distinguish different species of *Phallus* (Hemmes and Desjardin, 2009). The indusium may serve as a structure allowing crawling insects to climb up to the gleba, enticing insects that are not otherwise attracted by the odor (Baseia et al., 2006). The NCBI database^1^ lists more than 35 species in the genus *Phallus*. Stinkhorn mushrooms have unique characteristics but few morphological characteristics useful for classification. In contrast, the mitogenome is a reliable tool for eukaryotic phylogenetic analysis, and it has been previously used in taxonomic and evolutionary studies of the Basidiomycetes (Li et al., 2019d, 2020). The mitogenomes of *Phallus*, Phallaceae, and Phallales, have not been reported, and this has limited understanding of stinkhorn fungi evolution.

We sequenced, assembled, and annotated the mitogenomes of *P. indusiatus* and *P. echinovolvatus*. We compared the mitogenome size, gene content, gene arrangement, and repetitive sequences of these two species and evaluated the similarity and variability of their mitogenomes. The mitogenome size, base composition, gene arrangement, and the dynamic changes of introns of *Phallus* and previously sequenced species within the Basidiomycetes were compared. The combined mitochondrial gene datasets were assessed as molecular markers to determine phylogenetic relationships between *Phallus* and other Basidiomycete species. The mitogenomes of the two species provide a basis for advanced research on taxonomy, phylogeny, conservation genetics, and evolutionary biology of this genus.

## Materials and Methods

### Sampling and DNA Extraction

Fruiting bodies of *P. indusiatus* and *P. echinovolvatus* were collected from the main production areas in Yibin and Guangyuan, Sichuan, China. Dried *Phallus* fruiting bodies were used for DNA extraction. Total DNA was extracted using the fungal DNA Kit D3390-00 (Omega Bio-Tek, Norcross, GA, United States) according to the manufacturer’s instructions. The quality of extracted DNA was determined by Picogreen fluorescence detection and agarose gel electrophoresis. The qualified samples were used for further sequencing analysis.

### Sequencing, Assembly, and Annotation of the Mitogenomes

Purified DNA was used to construct sequencing libraries (insert size of 400 bp) following the Illumina sequencing protocol. Whole-genome shotgun sequencing was performed using the Illumina HiSeq 2500 Platform (Nanjing Personal Gene Technology Co., Ltd., Nanjing, China) to obtain 2 × 150 bp reads. We assembled all the reads using Velvet 1.2.03 software (Zerbino and Birney, 2008) with different Kmer values. The clean reads obtained were screened with bowtie2 (Langdon, 2015), using the mitogenomes of closely related species as references. We used SPAdes 3.9.0 software (Bankevich et al., 2012) to *de novo* assemble the mitogenomes and MITObim V1.9 (Hahn et al., 2013) to fill in the gaps between contigs.

We used the MFannot (Lang B.F. et al., 2007) and MITOS (Bernt et al., 2013) tools to annotate the two *Phallus* mitogenomes, both based on genetic code 4. Intron-exon boundaries in conserved genes were adjusted manually by sequence alignments with corresponding sequences, without introns, from known mitogenomes of closely related species. The open reading frame (ORF) genes were modified and predicted with the NCBI Open Reading Frame Finder (NCBI Resource Coordinators, 2018) and then annotated by BLASTX queries against the non-redundant NCBI database. The tRNA genes were predicted by the tRNAscan-SE 2.0 (Lowe and Chan, 2016) program. Graphical maps of the mitogenomes were drawn by OGDraw v1.2 software (Lohse et al., 2013).

### Sequence Analysis of the *Phallus* Mitogenomes

We used Lasergene v7.1 to analyze the base composition of the *Phallus* mitogenomes and calculated the AT skews and GC skews of their mitochondrial genes and whole genomes, where AT skew = (A − T)/(A + T) and GC skew = (G − C)/(G + C) (Wang et al., 2017). We used DnaSPv6.10.01 (Rozas et al., 2017) to calculate the synonymous (*Ks*) and nonsynonymous (*Ka*) substitution rates of 15 conserved PCGs in the two mitogenomes, and mega v6.06 (Caspermeyer, 2016) was used to analyze the genetic distance of these PCGs, with the Kimura-2-parameter (*K2P*) as the substitution model. Genomic synteny analysis of mitogenomes from representative species within the Basidiomycetes was conducted with Mauve v2.4.0 (Darling et al., 2004).

### Repetitive Elements Analysis

We searched the entire mitogenome of the two *Phallus* species by BLASTn searches against themselves to identify large intragenomic replications of sequences and interspersed repeats, with an *E*-value of <10^–10^. Tandem repeats were detected using the Tandem Repeats Finder (Benson, 1999) with default settings.

### Gene Order, Introns, and Comparative Mitogenomic Analysis

Gene orders of 15 conserved PCGs, as well as *rnl* and *rns* genes, in the mitogenomes of the two species were compared with those of 42 genera within Basidiomycota. RNAweasel algorithm (Lang B.F. et al., 2007) was used to determine the intron types. Introns on *cox1* genes of all 44 species were analyzed and classified into different position classes (Pcls) using the method described by Férandon et al. (2010). Pcls were named with different letters, and any undescribed Pcls were marked with adjacent letter subscript numbers. The mitogenome sizes, base composition, introns, PCGs, RNA genes, and Intergenic regions were compared among the mitogenomes of the 44 species of Basidiomycota.

### Phylogenetic Analysis

We constructed a phylogenetic tree based on 14 conserved PCGs from the two *Phallus* species and 42 genera in Basidiomycota, including genera in the Cantharellales (2), Gomphales (1), Hymenochaetales (8) Polyporales (4), Russulales (5), Boletales (2), Agaricales (19), and Sebacinales (1). We used one species from the Ascomycota as the outgroup. MAFFT v7.037 software (Abascal et al., 2010) was first used to align the single mitochondrial gene sequence, and then SequenceMatrix v1.7.8 software (Lanfear R. et al., 2017) was used to concatenate the aligned single genes into a combined matrix. Modelgenerator v851 tool (Keane et al., 2006) was used to determine the best-fit evolutionary model for the phylogenetic analysis. We used the Bayesian inference (BI) method for phylogenetic analysis based on the combined gene dataset in MrBayes v3.2.6 (Ronquist et al., 2012).

## Results

### Genome Features and PCGs of *Phallus* Mitogenomes

**FIGURE 1:**
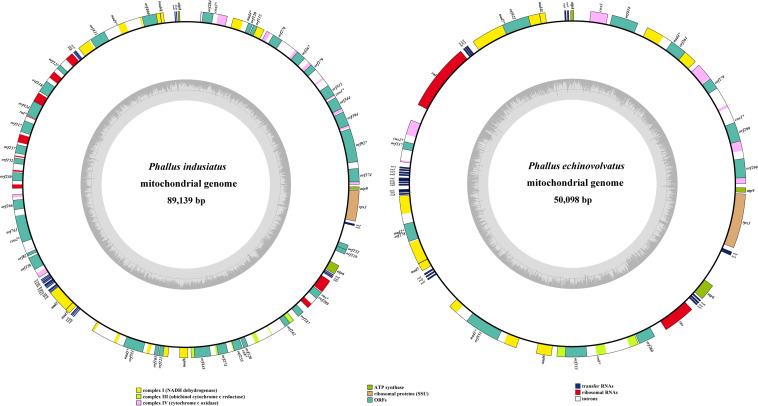
Circular maps of the mitogenomes of two *Phallus* species. The first nt of the cox1 gene was defined as nt 1 of the genome artificially. Various genes are represented with different color blocks.

The mitogenomes of *P. indusiatus* and *P. echinovolvatus* were both composed of circular DNA molecules, with sizes of 89,139 and 50,098 bp, respectively (Figure 1). The GC content of *P. indusiatus* (24.74%) was similar to that of *P. echinovolvatus* (24.30%; Table 1). Both the AT skews and GC skews of the two species are positive. The two mitogenomes contained 14 typical PCGs for energy metabolism and one *rps3* gene for transcriptional regulation. In addition to the conserved genes, we identified 36 ORFs in the *P. indusiatus* mitogenome, including 33 located within introns and three intergenic ORFs (Supplementary Table S1). The *P. indusiatus* mitogenome contains one ORF encoding reverse transcriptase, 32 ORFs encoding homing endonucleases with the GIY-YIG and LAGLIDADG domains, and two ORFs of unknown function. In contrast, only 11 ORFs were detected in the *P. echinovolvatus* mitogenome, including nine within introns and two between genes. The mitogenome of *P. echinovolvatus* contained a quinate/shikimate dehydrogenase gene, two PCGs with unknown function, and eight ORFs encoding homing endonucleases with the GIY-YIG and LAGLIDADG domains. We identified 33 introns in the mitogenome of *P. indusiatus*, distributed in *cox1* (9), *nad4* (1), *cox3* (1), *nad5* (3), *rnl* (7), *cox2* (3), *nad1* (3), *cob* (5), and *rns* (1). Only 10 introns were observed in the mitogenome of *P. echinovolvatus*, distributed in *cox1* (3), *nad4* (1), *nad5* (1), *cox2* (1), *nad2* (1), *nad1* (1), and *cob* (2). All of the detected genes in the mitogenomes of *P. indusiatus* and *P. echinovolvatus* were located on the sense strand.

**TABLE 1:** Comparison of two *Phallus* mitogenomes.

Item	Accession number	Genome size (bp)	GC content (%)	AT skew	GC skew	No. of non-intronic PCGs	No. of introns	Intronic ORFs	No. of rRNAs	No. of tRNAs
*P. indusiatus*	MT528240	89,139	24.74	0.0349	0.0863	18	33	33	2	24
*P. echinovolvatus*	MT528241	50,098	24.30	0.0121	0.0964	17	10	9	2	24

### rRNA Genes and tRNA Genes

The mitogenomes of *P. indusiatus* and *P. echinovolvatus* both contained two rRNA genes, a large subunit ribosomal RNA gene (*rnl*), and a small subunit ribosomal RNA gene (*rns*; Supplementary Table S1). A total of eight introns were observed in the rRNA genes of the *P. indusiatus* mitogenome. No introns were identified in the rRNA genes of the *P. echinovolvatus* mitogenome. The nucleotide lengths of *rnl* and *rns* genes in the *P. indusiatus* mitogenome were 5 and 1 bp longer than those of *P. echinovolvatus*, respectively.

The mitogenomes of *P. indusiatus* and *P. echinovolvatus* both contain 24 tRNA genes, ranging in size from 71 to 88 bp (Supplementary Table S1). The tRNA genes of the two *Phallus* mitogenomes clustered into six groups, which were located in the regions between *atp8* and *nad4L* (WD), *nad5* and *rnl* (NCR), *cox2* and *nad2* (AFYLMGMETIK), *nad3* and *nad1* (SQH), *rns* and *atp6* (VLR), and *atp6* and *rps3* (SP; Figure 1). The 24 tRNA genes encode all 20 standard amino acids, and all were folded into classical cloverleaf structures (Supplementary Figures S1, S2).

### Intergenic Regions and Mitogenome Composition

The two *Phallus* mitogenomes contained a common overlapping region (1 bp), found in filamentous fungi mitogenomes, located between *nad4L* and *nad5* (Supplementary Table S1). In addition, an overlapping region (41 bp) was found between *orf129* and *orf152_2* in the *P. indusiatus* mitogenome. We detected 13,055 and 10,936 bp intergenic sequences in the mitogenomes of *P. indusiatus* and *P. echinovolvatus*, respectively. The longest intergenic sequence (1,837 bp) of the *P. indusiatus* mitogenome was observed between *nad1* and *nad6*. In contrast, the longest intergenic sequence (1,619 bp) of *P. echinovolvatus* mitogenome was between *atp6* and *trnS* (tga).

**FIGURE 2:**
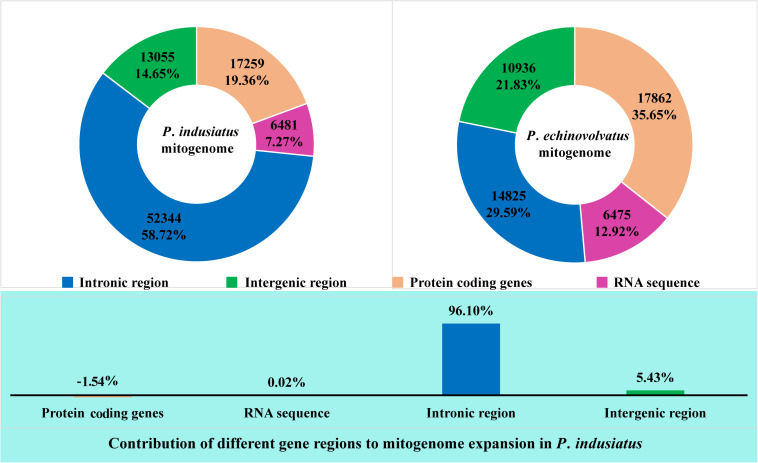
The intronic, intergenic, protein-coding, and RNA gene region proportions of the entire mitogenomes of two *Phallus* species. The bottom panel shows the contribution of different gene regions to the expansion of the *P. indusiatus* mitogenome. The value of bar chart is based on the ratio of differences in the protein-coding genes, RNA sequence, intronic region, and intergenic region between *P. indusiatus* and *P. echinovolvatus* mitogenomes to the difference of their entire mitogenomes.

An intronic region accounted for the largest proportion (58.72%) of the mitogenome of *P. indusiatus*, followed by the protein coding regions, which accounted for 19.36% of the entire genome (Figure 2). In contrast, the protein coding regions comprised the largest proportion (35.65%) of the *P. echinovolvatus* mitogenome. The intronic region accounted for 29.59% of the length of the *P. echinovolvatus* mitogenome. Similarly, the RNA coding regions and intergenic regions accounted for the smallest and second smallest proportion of the two *Phallus* mitogenomes, comprising 7.27–12.92% and 14.65–21.83% of the total mitogenome lengths. The *P. indusiatus* mitogenome is much larger (39,041 bp, 43.80% greater) than that of *P. echinovolvatus.* The intronic region contributed to 96.10% of the increased mitogenome size of *P. indusiatus* (Figure 2). The intergenic regions, the second largest factor, only contributed 5.43% to the size expansion of the *P. indusiatus* mitogenome. The results indicate that the intronic region was the most important factor related to the mitogenome expansion of the two *Phallus* species.

### Repeat Sequence Analysis

By comparing the whole mitogenomes against themselves via BLASTN search, we found four repeat sequences in *P. indusiatus* and two repeat sequences in *P. echinovolvatus* (Supplementary Table S2). The length of the repeat sequences in the two *Phallus* mitogenomes ranged from 58 to 1,343 bp, with pair-wise nucleotide similarities ranging from 70.29 to 92.26%. The longest repeat region of the *P. indusiatus* mitogenome was located between *nad5*-i1 and *rns*-i1. The largest repeat observed in the *P. echinovolvatus* mitogenome was 168 bp and was located in a free-standing ORF (*orf414*), *cox3*, and intergenic sequences around them. The repetitive sequences detected in *P. indusiatus* and *P. echinovolvatus* mitogenomes accounted for 5.29 and 0.90% of the entire mitogenomes, respectively.

We detected six tandem repeats in the mitogenome of *P. indusiatus* and seven tandem repeats in *P. echinovolvatus* (Supplementary Table S3). The tandem repeats observed in the two *Phallus* mitogenomes contained 2–6 copies, with lengths ranging from 5 to 24 bp. The longest and highest replication number tandem repeat sequences were observed in the *P. echinovolvatus* mitogenome. The tandem sequences accounted for 0.29 and 0.60% of the mitogenome lengths of *P. indusiatus* and *P. echinovolvatus*, respectively.

### Variation, Genetic Distance, and Evolutionary Rates of PCGs

**FIGURE 3:**
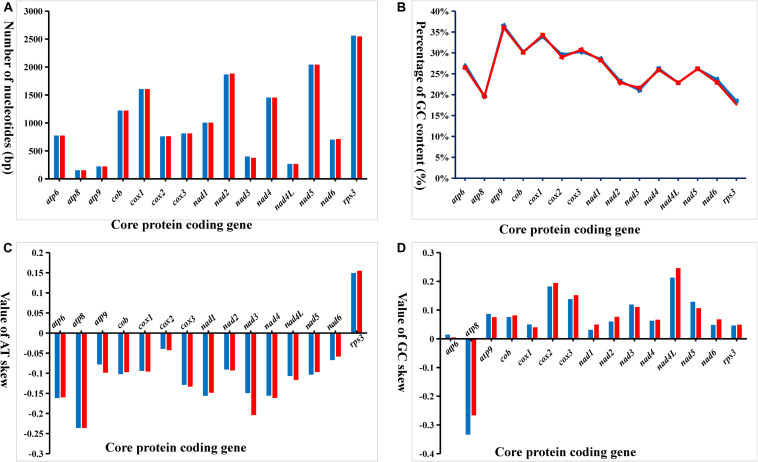
Variation in the length and base composition of each of 15 protein-coding genes (PCGs) between two *Phallus* mitogenomes. **(A)** PCG length variation; **(B)** GC content of the PCGs; **(C)** AT skew; **(D)** GC skew.

Variation in length of the 15 core PCGs in the two *Phallus* mitogenomes occurred in the *nad2*, *nad3*, *nad6*, and *rps3* genes (Figure 3). The length of *nad3* and *rps3* in *P. indusiatus* mitogenome was larger than that of *P. echinovolvatus*, with differences of eight and five amino acids, respectively. In contrast, the lengths of *nad2* and *nad6* in the *P. indusiatus* mitogenome were shorter than those of the *P. echinovolvatus*, by six and three amino acids, respectively. The GC contents of most PCGs (80%, 12 of 15) varied in the two *Phallus* mitogenomes. Among the 15 PCGs in the two *Phallus* mitogenomes, the GC content of *atp9* was the highest, and that of *rps3* was the lowest. The GC content of *rps3* had the largest difference between the mitogenomes of *P. indusiatus* and *P. echinovolvatus*. AT skews of most core PCGs in the two mitogenomes were negative, with the exception of *rps3*. Except for *atp8*, which has a negative GC skew, all PCGs-coding genes had a positive GC skew. The AT skews and GC skews varied between the two mitogenomes with the exception of *atp8*, indicating frequent base variation in the core PCGs of the *Phallus* mitogenomes.

**FIGURE 4:**
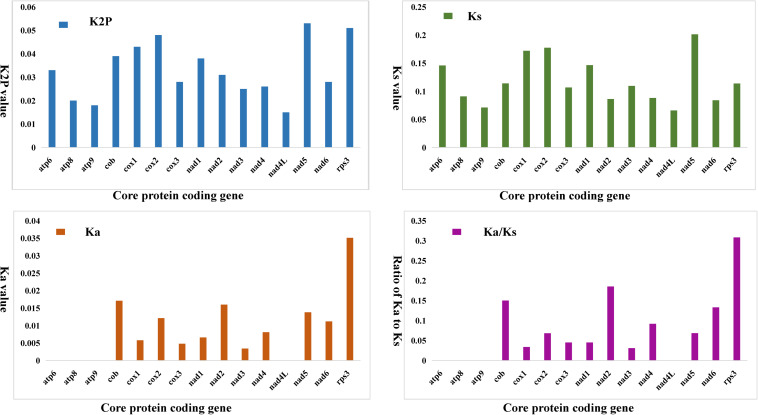
Genetic analysis of 15 protein-coding genes conserved in two *Phallus* mitogenomes. *K2P*, the Kimura-2-parameter distance; *Ka*, the mean number of non-synonymous substitutions per non-synonymous site; *Ks*, the mean number of synonymous substitutions per synonymous site.

We compared the *K2P* genetic distance of 15 conserved PCGs in the two *Phallus* mitogenomes. The *K2P* genetic distance of *nad5* was the largest (Figure 4), followed by *rps3* and *cox2*. This indicated that these genes had the largest differences in the two *Phallus* species. The *nad4L* had the smallest *K2P* genetic distance among the 15 conserved PCGs between the two mitogenomes, suggesting that this gene is relatively conserved in the two species. Among the 15 conserved PCGs, the *nad5* gene had the highest synonymous substitution (*Ks*) rate, while *nad4L* has the lowest *Ks* rate between the two species. The *rps3* gene exhibited the highest non-synonymous substitution rate (*Ka*), while *atp6*, *atp8*, *atp9*, and *nad4L* had the lowest *Ka* values. The *Ka*/*Ks* values of all 15 conserved PCGs were less than 0.35, suggesting that the two species have undergone a relatively pure evolutionary process.

### Mitochondrial Gene Arrangement in Basidiomycota Species

**FIGURE 5:**
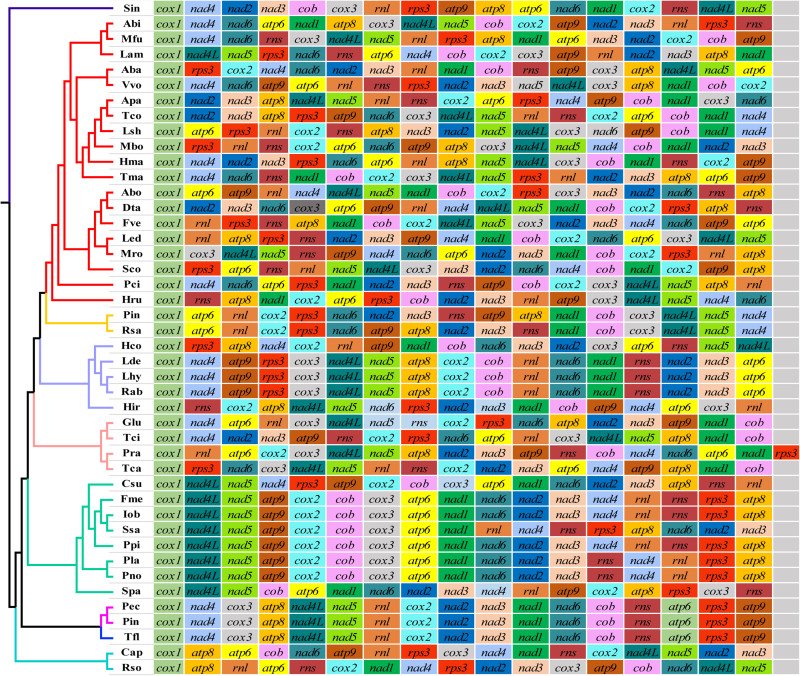
Mitochondrial gene arrangement analyses of 44 Basidiomycete species. Genes are represented with different color blocks. All genes are shown in order of occurrence in the mitochondrial genome, starting from *cox1*. Fourteen core protein-coding genes, one *rps3* gene, and two rRNA genes were included in the gene arrangement analysis. Species and NCBI accession number used for gene arrangement analysis in the present study are listed in Supplementary Table S5.

A total of 36 different gene rearrangement groups were observed in the mitogenomes of 43 genera of Basidiomycota (Figure 5), indicating that Basidiomycota mitogenomes have undergone large-scale gene rearrangements. The gene order of the two *Phallus* mitogenomes was identical, and also identical to that of *Turbinellus floccosus*, which belongs to the Gomphales order. In the other seven orders of Basidiomycota studied, the mitochondrial gene order was inconsistent between any of two orders. Similarly, of the 28 families studied, there was no identical arrangement of mitochondrial genes between any two families except the Phallaceae and Gomphaceae, which belong to the Phallales and Gomphales, respectively. At the family level, the gene orders were highly variable except for some genera in the Hymenochaetaceae and Russulaceae.

**FIGURE 6:**
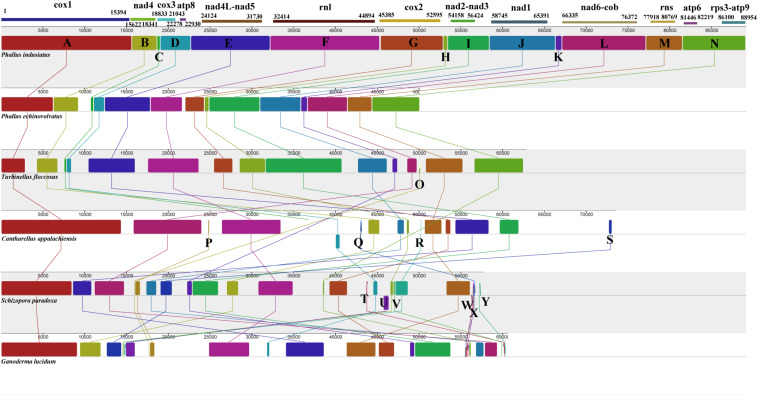
Mitogenome synteny among six Basidiomycetes species. Twenty-three homologs regions (A–Y) were identified among the six mitogenomes. The sizes and relative positions of the homologs fragments varied across the mitogenomes.

We analyzed the mitogenome homology of two *Phallus* species and four species in the orders Gomphales, Cantharellales, Hymenochaetales, and Polyporales. The six mitogenomes were divided into 23 homologs regions (Figure 6). The *P. indusiatus* and *P. echinovolvatus* mitogenomes contained the same homologs regions (A–N), which are also present in the *T. floccosus* mitogenome in the same order. The *T. floccosus* mitogenome has an extra region “O” that is not present in the two *Phallus* species. In addition to *P. indusiatus*, *P. echinovolvatus*, and *T. floccosus*, the three other species had 10 new homologs regions (P-Y), and the order of the homologs regions was different. This indicates significant differences in the type and arrangement of genes in these species.

### Analysis of Mitogenome Size Variation in Basidiomycota Species

The mitogenome sizes of the Basidiomycota mitogenomes varied greatly, ranging from 37,341 bp for *Amanita basii* to 235,849 bp for *Rhizoctonia solani*. We analyzed the mitochondrial genetic composition of 43 genera of Basidiomycota, including the number, length and proportion of introns, PCGs, RNA genes, and intergenic regions, to identify variation in their genome size (Supplementary Table S4). Though there was great variation in the mitogenome sizes among the different species, the numbers and lengths of RNA genes and conserved protein-coding genes (PCGs) were conserved. *Rhizoctonia solani*, with the largest mitogenome (235.8 kb), had the longest lengths of introns (88.3 kb) and intergenic regions (110.6 kb). *Pyrrhoderma noxium* had the second largest mitogenome (163.4 kb), containing the largest number of introns (61). In most species with larger mitogenome sizes, the length of their introns and intergenic regions was longer than that of other species and accounted for a large proportion of the total mitogenome. This indicated that these are important factors contributing to mitogenome length variation. *P. indusiatus* had an intermediate-size mitogenome (89.1 kb) with an intron length of 52.3 kb, accounting for 58.7% of the total mitogenome. The proportion of introns in the *P. indusiatus* mitogenome was the largest of all Basidiomycete species. *P. echinovolvatus* had a relatively small mitogenome (50.1 kb), with intron lengths reaching 14.8 kb (29.6% of its entire mitogenome), which is much higher than that of other species of similar size. Introns appear to play an important role in the composition of the mitogenome of *Phallus.* The number and length of free-standing ORFs varied in all the species analyzed, regardless of the mitogenome size, suggesting that free-standing ORFs have undergone many changes in species evolution.

### Intron Dynamics of *cox1* Gene in Basidiomycota Species

**FIGURE 7:**
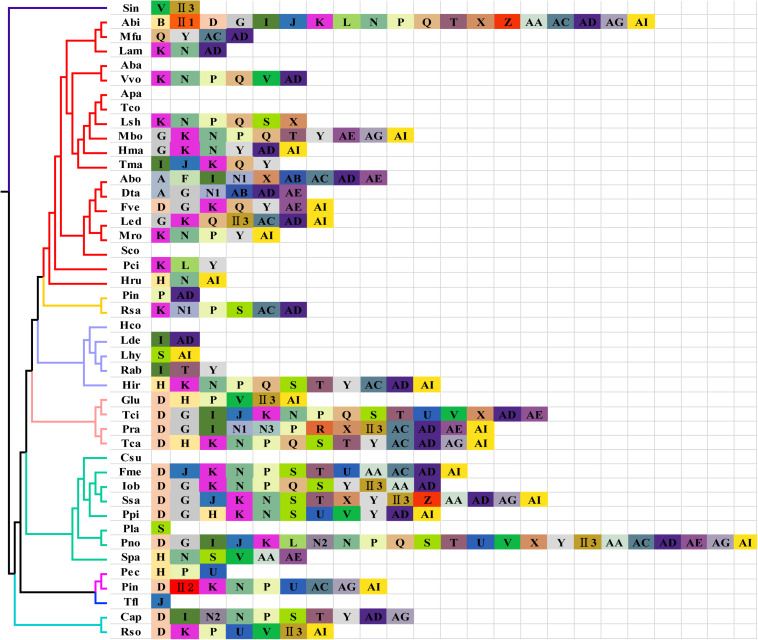
Position class (Pcl) information of cox1 gene of 44 Basidiomycete species. The same Pcl (orthologs intron) is represented by the same letter. The phylogenetic positions of 44 Basidiomycete species were established using the Bayesian inference (BI) method based on a 14 conserved mitochondrial gene set. N1–N3 represent the newly detected Pcl in this study; II in the figure shows that the intron belongs to group II introns. Species IDs are shown in Supplementary Table S5.

In eukaryotes, different introns can be classified based on their specific insertion site. Introns at the same insertion site are assigned to the same position class (Pcl), and their nucleotide sequences tend to be relatively similar. Intronic ORFs on their introns are also relatively similar (Férandon et al., 2010). Of the 44 Basidiomycete species analyzed, 38 species had introns in the *cox1* gene, for a total of 283 introns, including 273 group I introns and 10 group II introns (Figure 7). The highest number (23) of *cox1* introns was found in *P. noxium*. We detected 34 types of Pcls in 44 Basidiomycete species, six of which were new loci and have not been previously reported. Pcl K was the most common intron type and was found in the *cox1* genes of half of the species analyzed, followed by Pcls N, P, Y, and D, which were found in *cox1* genes of >30% of species. These data indicate that these introns are common intron types in the Basidiomycetes. The *cox1* genes of two species of the genus *Phallus* share Pcls P, and U, but *P. echinovolvatus* lacks Pcls D, II2, K, N, AC, AG, and AI, and *P. indusiatus* lacks Pcl H. This suggests that frequent intron gain and loss events have occurred in the mitogenomes of *Phallus*.

**FIGURE 8:**
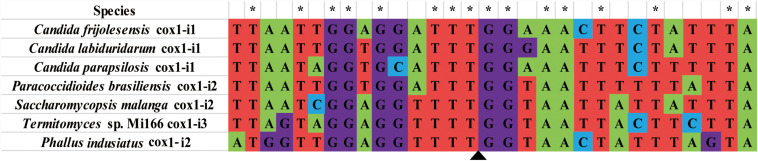
Comparison of 15 bp sequence upstream and downstream of the insertion site of Pcl II2. Probable insertion site is shown by the triangle. Asterisks represent base pairs that are in the same corresponding position. Sequences were aligned by Clustal W in MEGA v6.06.

Among the 283 introns detected in the cox1 gene of 44 Basidiomycete species, Pcl II2 was only found in the *P. indusiatus* mitogenome. We compared the sequence of the Pcl II2 intron and the intronic ORF in the NCBI database and found that six fungal species had the same type of intron. Pcl II2 was also detected in Stramenopiles species, such as *Halamphora calidilacuna* (57.7% aa similarity) and *Psammoneis japonica* (53.1% aa similarity). The nucleic acid sequences (15 bp of upstream and downstream) of insertion sites of Pcl II2 were assessed in this study. The results showed that 16 out of 30 base pairs were identical in seven fungal mitogenomes, and the insertion site of Pcls II2 was relatively conservative (Figure 8). Pcl II2 was inserted into the downstream of base TTT and upstream of GG. The results could be useful for accurate identification of Pcl II2 and the annotation of mitochondrial genes with multiple introns.

### Phylogenetic Relationship Analysis

**FIGURE 9:**
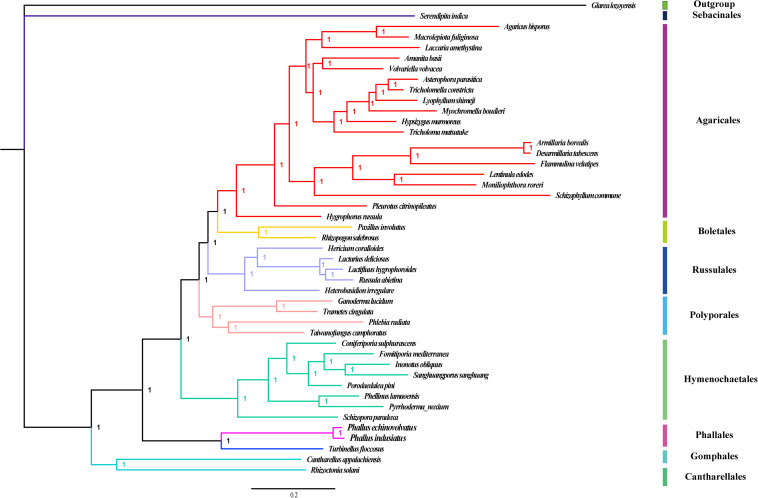
Bayesian phylogenies of 44 fungal species using individual genes including each of 14 conserved protein-coding genes. *Glarea lozoyensis* was used as an outgroup. Support values are Bayesian posterior probabilities. Species and NCBI accession number used for phylogenetic analyses are provided in Supplementary Table S5.

Bayesian inference was used to establish the phylogenetic relationship among 42 genera from the Basidiomycetes, as well as the two novel *Phallus* species. *Glarea lozoyensis* from Ascomycota was used as an outgroup. The number of total nucleotides obtained from the concatenated alignment of the gene sets used in phylogenetic analyses was 23,293. We constructed a phylogenetic tree based on a combined 14 conserved PCGs set and obtained a stable evolutionary tree topology (Figure 9) with all of the recovered clades well supported (Bayesian posterior probability; BPP = 1). Forty-four species of Basidiomycetes were clustered into eight groups, mainly corresponding to the orders Cantharellales, (Gomphales + Phallales), Hymenochaetales, Polyporales, Russulales, Boletales, Agaricales, and Sebacinales. The genera *Phallus* and *Turbinellus* from the orders Phallales and Gomphales were clustered on the same branch and were different from other orders of Basidiomycetes. This indicated that these two orders are closely related. *Serendipita indica* of the Sebacinales order was clustered on a distinct branch of the evolutionary tree, suggesting that it is distantly related to other Basidiomycete species. The mitogenomes of Agaricales are the most analyzed group in Basidiomycetes, and their phylogenetic relationships were consistently recovered as [Hygrophoraceae + (Pleurotaceae + (Schizophyllaceae + Marasmiaceae+Omphalotaceae + Physalacriaceae + Physalacriaceae + (Tricholomataceae + Lyophyllacea + Pluteaceae + Amanitaceae + (Hydnangiaceae + Agaricaceae))))].

## Discussion

The mitogenome is often used in phylogenetic and evolutionary studies of eukaryotes due to its small genome size, conserved gene sequence, low level of recombination, and numerous molecular markers (Zhang et al., 2017; Ramos et al., 2018). Compared to animals, research on the mitogenomes of fungi is infrequent (Song et al., 2020). The development of next-generation sequencing technology has enabled research on fungal mitogenomes (Li et al., 2019c). However, for some important species, such as stinkhorn mushrooms, the lack of mitogenome research has hindered understanding of their evolution. We analyzed the mitogenomes of two species of bamboo mushrooms and present the first report of mitogenomes in the genus *Phallus*. The mitogenome length of *P. indusiatus* was about 1.8 times longer than that of *P. echinovolvatus*. The significant difference in the length of mitogenomes between these two *Phallus* species illustrates interspecific variability of mitogenome size. Substantial size differences also exist within many other genera of the Basidiomycetes (Li et al., 2018b). The other 42 genera of Basidiomycota studied varied greatly in mitogenome size and composition (Supplementary Table S4). Variation in introns, intergenic regions, and free-standing ORFs were the three most important factors and had a major influence on the mitogenome size of these species. The influence of these factors on mitogenome length has been confirmed by other research on fungal mitogenomes (Himmelstrand et al., 2014; Chen et al., 2019). Introns accounted for 58.7% of the length of the *P. indusiatus* mitogenome, and this was greater than any of the other 43 species. The intronic region contributed 96.10% of the increased mitogenome size of *P. indusiatus*. The size and proportion of introns in the *P. echinovolvatus* mitogenome were much larger than those in other species with similar mitogenome sizes. These data suggest that introns are the most important factor in the variation of mitogenome size in *Phallus*.

The mitogenomes of eukaryotes have independent evolutionary origins that have developed over long time spans (Thielsch et al., 2017). During natural selection, some mitochondrial genes are transferred to nuclear genomes, while others are preserved (Adams and Palmer, 2003). These episodic mitochondrial gene transfer events complement the function of the nuclear genome (Adams and Palmer, 2003). Genes retained in the mitogenome have corresponding advantages, such as the production of hydrophobic proteins in the mitochondria to avoid long-distance transport from the nucleus and the maintenance of mitochondrial structure (Allen, 2015; Björkholm et al., 2015). The two *Phallus* mitogenomes preserved a complete set of 15 conserved PCGs (*atp6*, *atp8*, *atp9*, *cob*, *cox1*, *cox2*, *cox3*, *nad1*, *nad2*, *nad3*, *nad4*, *nad4L*, *nad5*, *nad6*, and *rps3*) for energy metabolism and transcriptional regulation. All of the Basidiomycetes analyzed retained these genes, unlike the Ascomycetes where gene loss events have occurred more frequently (Wolf and Giudice, 1988). The differences in length and genetic distances of some conserved PCGs in the two *Phallus* mitogenomes suggest that these genes have undergone different rates of evolution (Korovesi et al., 2018). The low *Ka/Ks* values of conserved PCGs also revealed the conservation of the mitogenomes of the two *Phallus* species. In addition, there are 36 ORF genes and 11 ORF genes distributed in *P. indusiatus* and *P. echinovolvatus* mitogenomes, most of which (40 of 47) encode the homing endonuclease, and four ORF genes have an unknown function. A variety of ORF genes are distributed in the mitogenome of fungi, and the origin and function of many ORF genes has not been determined (Duò et al., 2012). More research on the mitogenome of the *Phallus*, and related species, is needed to characterize the origin and function of these ORF genes.

The GC content of fungal mitogenomes varies, depending on selection, base mutation bias, and reconstruction-related DNA repair bias (Li et al., 2018c). The GC content of *P. indusiatus* is similar to that of *P. echinovolvatus.* However, the GC content of most of the core PCGs in their mitogenomes varied, indicating that their mitogenomes have undergone evolutionary changes. Similarly, AT skew and GC skew have been used as indicators of species evolution (Yuan et al., 2015). The negative or positive AT skews and GC skews indicate asymmetry of nucleotide composition between the two strands, with one being rich in A and C, and the other being rich in T and G. However, mitochondrial genes may be located on direct or revised strands, leading to a lack of scientific significance in data analysis. In this study, all of the detected genes in the mitogenomes of the two *Phallus* species were located on the direct strand. Their mitogenomes contained positive AT skews and GC skews. However, the AT skews of 14 core protein genes in *Phallus* mitogenome were negative. In addition, the AT skew and GC skew of almost all PCGs in the *P. indusiatus* and *P. echinovolvatus* mitogenomes varied. If there is no mutation or selection bias, each base in the complementary DNA strand should exist at approximately equal frequencies according to the second parity rule (Chen et al., 2014). The unique A–T and G–C biases in the mitogenomes of these two *Phallus* species indicate that they have undergone unique genetic mutations and/or selection.

Protein-coding genes, RNA genes, and repetitive sequences can all lead to rearrangement of eukaryote mitogenomes. These are important factors in studies of the origin and evolution of eukaryotes (Aguileta et al., 2014; Li et al., 2018a). Mitochondrial genes in animals have low rates of change compared to those in plants and fungi. A number of gene rearrangement models have been established to reveal the rearrangement of mitogenomes in animals (Perseke et al., 2008). Many mitochondrial gene rearrangements have also been found in Basidiomycetes, even in congeneric species (Li et al., 2019b). In the present study, 36 gene arrangement types were found in 43 different Basidiomycete genera, and no identical gene arrangement types were found in 26 families within six orders. This suggests that most Basidiomycetes have undergone substantial rearrangement of their mitochondrial genes during evolution. However, the mitogenomes of the two *Phallus* species in this study shared an identical mitochondrial genetic arrangement with *T. floccosus* from a different order. The homologs regions of *Phallus* were almost identical to those of *T. floccosus*. Using morphological characteristics and multiple molecular markers of nuclear genes, Hosaka et al. (2006) classified the orders Gomphales and Phallales into a new subclass (Phallomycetidae). It is possible that the fungal species of Phallomycetidae underwent a similar evolutionary process.

Intron sequences within the gene coding regions are widely distributed in the mitogenome of fungi. Most are type I introns, and these are mainly distributed in the *cox1* gene (Lang B.F. et al., 2007; Férandon et al., 2010). Introns are considered to be mobile elements associated with genetic loss or gain events, and they are distributed in different species of eukaryotes (Cusimano et al., 2008). In fungi, introns have retained their ability to spread to intron-free target sites, but can also be lost again through fortuitous deletion or conversion events (Repar and Warnecke, 2017). We detected 34 Pcls in 44 Basidiomycete species. Six Pcls were not reported, indicating a wide variety of intron types in Basidiomycetes. Pcl K was the most common intron type, and it is possible that it existed in ancestors of the Basidiomycetes and has been retained during evolution for a specific, and important, role. The mitogenomes of the two *Phallus* species had various Pcls types, suggesting that they have experienced frequent loss and gain events during evolution. Among the mitogenomes of 44 Basidiomycetes, Pcl II2 was only detected in the mitogenome of *P. indusiatus.* The Pcl II2 was observed in distantly related species (*H. calidilacuna* and *P. japonica*), suggesting a horizontal gene transfer event. Introns containing homing endonucleases often have unique insertion sites that can be used as a tool for intron recognition and gene editing (Férandon et al., 2010). We analyzed the mitogenomes of fungi with the same Pcl II2, and the possible insertion sites of Pcl II2 were TTT upstream and GG downstream. This provided a basis for the annotation of other multi-intron species containing Pcl II2.

Due to the variety of fungi, morphological similarity among species, and the difficulty in obtaining nuclear genes for multi-gene identification, accurate identification of many fungal species is difficult. The mitogenome is often used in phylogenetic studies of fungi due to its independent genetic characteristics relative to nuclear genomes and abundant molecular markers (Liang et al., 2017). The mitogenomes of many Basidiomycete species have been studied, and this has provided a basis for taxonomic analysis (Nieuwenhuis et al., 2019). However, there are no mitochondrial molecular markers for phylogenetic and population analysis of the Phallales. Combined genetic datasets, such as *LSU*, *EF-1a*, and *RPB2* genes, have been used for phylogenetic studies in Phallales, and new taxa have been identified (Hosaka et al., 2006). The mitogenome data of *Phallus* now allow for the phylogenetic analysis of Phallales. We constructed a phylogenetic tree based on a set of 14 conserved PCGs in *Phallus* and 42 other Basidiomycete genera. The high support rate (BPP = 1) suggested that these mitochondrial genetic data could be used as molecular markers. The mitochondrial phylogenetic relationships also confirm the accuracy of some generic reclassifications. One example is *Tricholomella*, which belongs to Lyophyllaceae and is distinct from *Tricholoma* of the Tricholomataceae (Kalamees, 1992). The two *Phallus* species have a close phylogenetic relationship with *T. floccosus*, in the subclass Phallomycetidae. Additional mitogenomes are needed to assess the origin and evolution of other fungal subclasses.

## Data Availability Statement

The datasets presented in this study can be found in online repositories. The names of the repository/repositories and accession number(s) can be found in the article/Supplementary Material.

## Author Contributions

CC and DL conceived this study. CC, JW, and QL performed the experiments. CC, JW, QL, and RF analyzed the data, prepared the figures, and drafted the manuscript. XJ and WH participated in analysis of preliminary data. CC wrote the manuscript. JW, QL, and DL provided suggestions for the research and critically revised the manuscript. All authors read and approved the final version of the manuscript.

## Conflict of Interest

The authors declare that the research was conducted in the absence of any commercial or financial relationships that could be construed as a potential conflict of interest.

## Footnotes

1 https://www.ncbi.nlm.nih.gov/Taxonomy/Browser/wwwtax.cgi?id=146780

## Supplementary Material

Supplementary Figure 1 Putative secondary structures of the 24 tRNA genes from *P. indusiatus* mitogenome. The tRNAs are labeled with the abbreviations of their corresponding amino acids. The tRNA arms are illustrated as for trnR.

Supplementary Figure 2 Putative secondary structures of the 24 tRNA genes from *P. echinovolvatus* mitogenome. The tRNAs are labeled with the abbreviations of their corresponding amino acids. The tRNA arms are illustrated as for trnR.
