# Supplementary figures and images for: Mitogenomes of Two Phallus Mushroom Species Reveal Gene Rearrangement, Intron Dynamics, and Basidiomycete Phylogeny
Source: Front Microbiol. 2020 Oct 23;11:573064. doi: 10.3389/fmicb.2020.573064 (PMC7644776; doi:10.3389/fmicb.2020.573064)

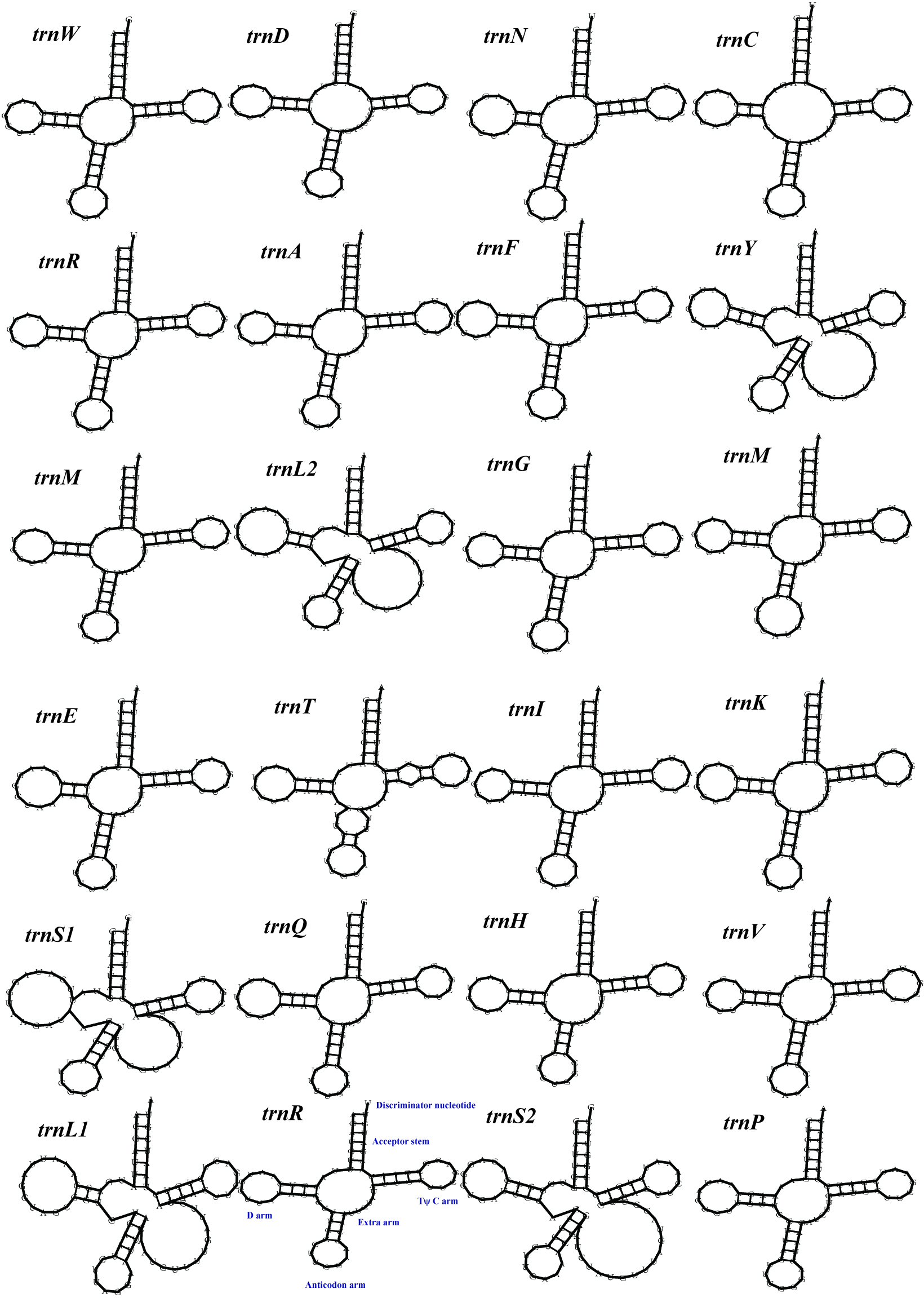

Supplement: Supplementary Figure 1 — Putative secondary structures of the 24 tRNA genes from P. indusiatus mitogenome. The tRNAs are labeled with the abbreviations of their corresponding amino acids. The tRNA arms are illustrated as for trnR. [file Image_1.TIF]

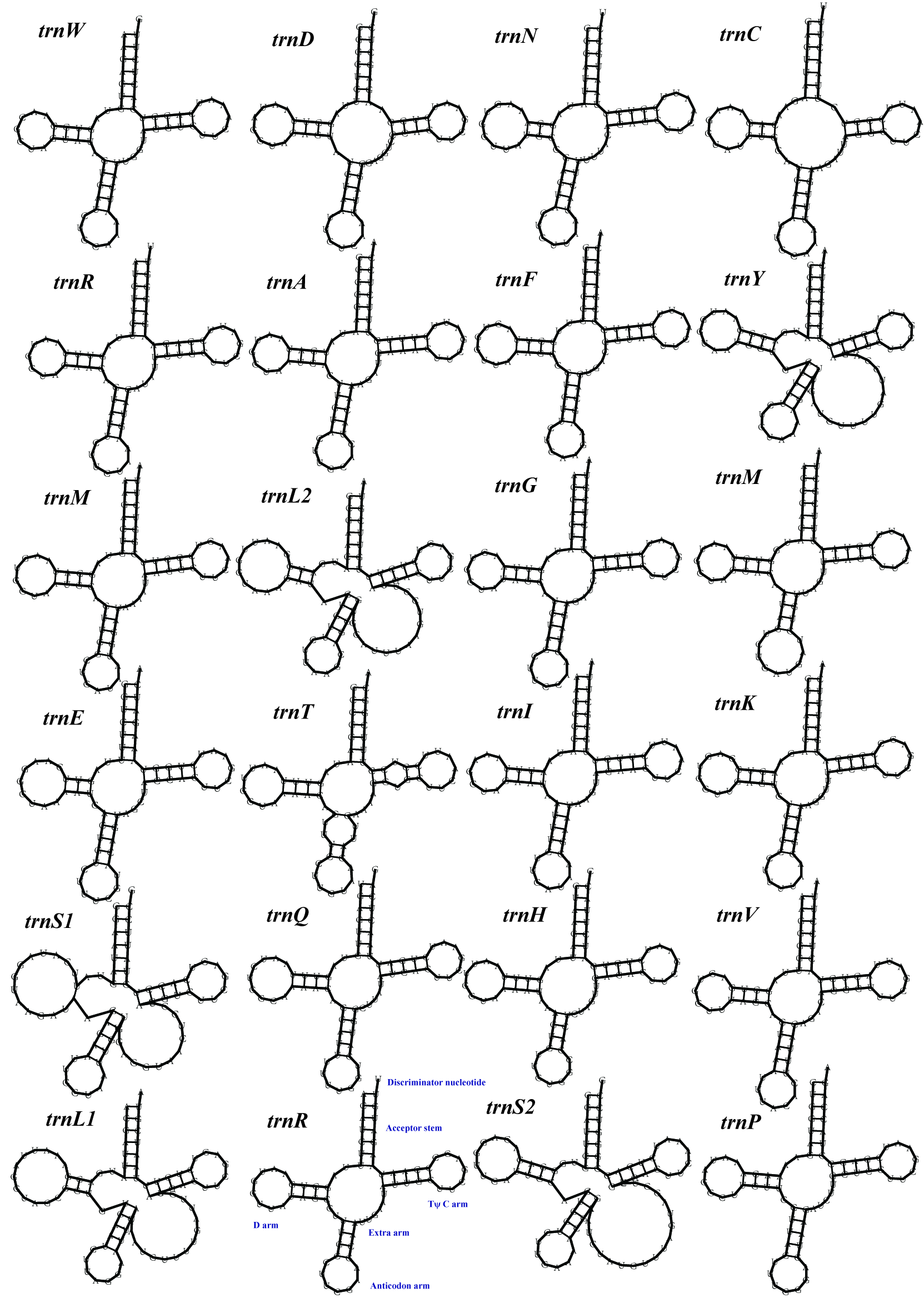

Supplement: Supplementary Figure 2 — Putative secondary structures of the 24 tRNA genes from P. echinovolvatus mitogenome. The tRNAs are labeled with the abbreviations of their corresponding amino acids. The tRNA arms are illustrated as for trnR. [file Image_2.TIF]
